# Deformities of Spine: Posterior Angular Curvature

**Published:** 1875-10

**Authors:** Lewis A. Sayre


Deformities of the Spine: Posterior Angular Curvature.—*By Lewis A. Sayre, M.D.*—Deformities of the spine may be a consequence of disease, and the important point in their study is to arrive at the pathological changes that have given rise to them. Of these deformities there are two: 1. The one known by the name of Pott's disease, or posterior angular curvature, in which there is distinctive inflammation of the bones, accompanied with loss of substance in the bodies of the vertebræ and intervertebral substance; 2. The deformity known as lateral rotary curvature of the spine, in which there is no disease of the bones, but the distortion depends entirely upon irregular muscular contraction. . . .

The posterior angular curvature, or Pott's disease, will first engage our attention. . . . It may occur at any period of life, but is much more likely to occur in childhood, and especially in those children who are reckless and careless, and expose themselves to all sorts of accidents. It also occurs more frequently among boys than among girls, because they are more exposed to accidents; whereas, the lateral curvature is seen more frequently among girls. With regard to this affection, I have arrived at the conclusion, based upon an accurate and carefully recorded experience, that it is produced almost always, if not always, by some injury to the bone, and is hence *traumatic* in its nature.

By the profession in general Pott's disease, above all others, has been considered as essentially of strumous origin; as depending upon a tuberculous diathesis, and not occurring unless constitutional dyscrasia is present; but, in my own judgment, it much more frequently depends upon some injury than upon any constitutional condition. . . . The accidents which produce this disease are usually concussions and blows. . . .

The *symptoms* of this disease vary according to its location in the spinal column. When it has advanced far enough to produce a deformity, there is usually no difficulty in diagnosis. . . If the disease is situated in the cervical region, long before any distortion appears the patient will complain of difficulty in swallowing; many have a choking sensation as if there was a string around the neck; difficulty about the larynx, producing an irritable and continued cough; pain in the thorax, etc. . . .

When the pain is in the dorsal region the patient very often complains of pain in the lower part of the chest and upper part of the abdomen; also a constricting sensation, as if a band was around the body; complains more or less of indigestion and flatulence, and may have been treated for dyspepsia. He may also complain of pain in the chest, pain about the heart, and perhaps may have been treated for rheumatism.

Again, when the disease is lower down in the spinal column, he may have a sense of constriction about the abdomen, may suffer from constipation and flatulence, and perhaps have been treated for worms.

When the disease is still lower in the spine, the leading symptoms may be those referable to the bladder and rectum. The *chief* symptom in the case may be a frequent desire to pass the urine. Then the patient may also suffer from streaking pains down the thighs.

When such symptoms are present and they cannot be explained by the presence of some well-recognized disease, always go back to the point where the nerves distributed to these regions make their exit from the spinal canal, and carefully examine the bony structures which surround them. . . .

Every joint of the lower extremities is bent for the purpose of preventing any concussion from affecting the bodies of the vertebræ. The chin is made to project; the shoulders become elevated; and it is impossible for the child to stand upright and receive any concussion whatever which may be communicated to the bodies of the bones without suffering pain. The muscles of the back are held rigid in order to prevent any movements of the bodies of the vertebræ upon each other. The child is unable to stoop down and pick up any object upon the floor; but, if asked to do so, he begins by bending his hips, and then his knees, and finally reaches the object by squatting down to it. These patients never bend the back, for bending the back presses the bodies of the vertebræ together and gives rise to pain; consequently all the movements of the child are directed in such a manner as to prevent any motion in the spinal column.

When walking about the room the child will reach with his hands from one object to another. . . . If he cannot obtain any support by catching hold of various articles within reach, he will rest his hands upon his thighs, in order to transmit the weight of the head and shoulders through the legs to the ground, thereby giving them support without bearing upon the diseased vertebræ. . . .

Strip the child naked, and lay him across your lap, face down, with the arms over one thigh and the legs over the other, and then gradually separate your thighs. When that is done the first thing you will notice, probably, will be that the child takes a long breath, a long-drawn sigh of relief. . . . As long as the child is held in that manner he will be perfectly comfortable, and breathe easily, if you do not carry the extension so far as to produce reflex muscular contraction. Then close the limbs again, and the muscles are at once excited to contract, and the child again begins his short, catching respiration. . . .

Now, all this can be done when the disease is in the anterior part of the *bodies* of the vertebræ, or in the intervertebral disks; but it may be, in the case which you are examining, that the anterior bodies of the bones and the disks have not yet become involved, and yet the child is suffering from Pott's disease; for, when the dorsal portion of the spinal column is affected, the disease does not always expend itself upon the anterior portion of the bodies of the vertebræ at first, but the part most extensively involved may be upon the sides of the vertebræ, where they form a junction with the ribs. . . .

You may be able to press the spine down without producing pain; percuss the spine without producing pain, and the spinal column may apparently be straight, all of which might lead you to the conclusion that it is not diseased; but pressure upon the ribs, which will bring their heads in contact with the articulating facets, gives the patient pain, and at once you have evidence of diseased vertebræ. . . .

The fact that pressure can be made over the spinous processes without causing pain is regarded by many as evidence that no disease of the bones is present. But it is the anterior portion of the body of the vertebræ that is affected, and, when these begin to give away, the spinous processes begin to stick out, and by crowding upon them we remove the pressure from the diseased surfaces, and consequently the suffering of the patient is diminished. . . .

I simply wish to make these points: that it is the result of injury in almost all cases; that this injury is followed by inflammatory action; that it can be diagnosticated by making extension and counter-extension upon the spine, and by pressure upon the sides of the vertebræ; also by symptoms referable to the distal extremities of the nerves involved in the disease, long before the deformity is produced. . . .

In the earlier stages (and it is during this period that treatment is most important) there is nothing which can compare with *rest*, absolute and complete, in the horizontal posture. . .

If there is tenderness along the spine, or any evidences of active inflammation, ice along the spine, by means of ice-bags placed upon either side opposite the seat of the disease, will be of the greatest service. If the pain is acute, a half-dozen leeches may be applied, and repeated every eight or ten days, and then followed by the ice-bags. . . .

Blisters, issues and setons, applied for the purpose of keeping up a long-continued counter-irritation and discharge, are positively injurious, and under no circumstances whatever should such measures be adopted. In the first place the child is already sufficiently disturbed and prostrated by the pain attending the disease, without tormenting him any more by agents which, from their very nature, will produce pain; and he is also already sufficiently emaciated, without establishing a suppurative process to him more so.

Rest in the horizontal posture, and continued until you can bring the diseased surfaces of bone together, without producing pain, is the only safe rule to guide you in giving the patient permission to assume the upright posture. When he is permitted to assume this posture, it must always be attended by some artificial support which shall remove all pressure from the bodies of the vertebræ. . . .

The idea involved in the construction of some instruments, namely, that of lifting the bodies of the vertebræ apart by placing the belt about the hips, and a support under the arms, is simply absurd, because the mobility of the scapulæ is so great that they can be lifted up, as far as the endurance of the patient will permit, without relieving the weight of the body. . . .

If you are not able to obtain any of the apparatus described, you may take a piece of ordinary sole-leather, dip it into cold water until it becomes perfectly soft and flexible, and, after the child has been straightened out as much as can be done with safety, mould it to the body, and secure it by means of a roller-bandage.

Again, they may be dressed with plaster of Paris, as you would a fracture. . . .

At first I was afraid that the thoracic compression would interfere with respiration, and therefore divided the cuirass in the median line as soon as the plaster was set; I then secured the lower or pelvic portion with a firm, non-elastic roller, and the upper or thoracic portion with *elastic* bands to allow more free lateral expansion of the chest.

Practical experience, however, has demonstrated that this is not necessary, but on the contrary is injurious, particularly if the disease involves the sides of the bodies of the dorsal vertebræ, and that the complete circling of the thorax in the immovable plaster bandage in these cases gives the greatest relief, as by this means the ribs are held absolutely motionless, and the respiration is compelled to be diaphragmatic and abdominal. When the thorax is thus firmly secured, the anus and perineum will rise and fall synchronously with the diaphragm, and the respiration be carried on without difficulty, as long as these parts are free from pressure. Pressure upward against these parts with the hand produces a feeling of suffocation. It is therefore necessary, when the thorax is thus secured, that the patient should sit upon a chair with a hole in the seat, like a close-stool, or use an inflated India-rubber ring, like the ordinary life-supporter.—*New York Medical Journal.*

Camphorated Phenol.—Bufalini describes the combination of camphor and carbolic acid.

If equal parts of carbolic and camphor be dissolved in alcohol, and the mixture be allowed to stand for 13 hours, a yellowish, oily stratum arises to the surface. This will not mix with the water or liquid, nor is the camphor precipitated by the alcohol. This substance is called camphorated phenol. It is best prepared as follows:

One part of carbolic acid and two of camphor are mixed in a vessel and allowed to stand for some hours. A reddish-yellow oily liquid will be formed, which is to be purified by washing with water. The properties of this combination are reddish-yellow color, oily appearance, smell of camphor, insoluble in water, but soluble in alcohol and ether. From considerable experience in its use, Bufalini concludes:

(1) Camphorated phenol produces the same effects as carbolic acid, but is less dangerous. It may be used both internally and externally, viz., in enteric fever, etc.

(2) It has the power of modifying unhealthy wounds and of destroying the parasites which are present in certain diseases, as septicæmia, typhoid fever, etc.

(3) The medical use of camphorated phenol is to be preferred to that of carbolic acid, as the former does not present the disadvantages of the latter.

(4) Camphorated phenol, when applied to wounds, does not irritate them or act as a caustic or disorganizing substance on them, and may be used in large doses, without producing symptoms of poisoning.—*The Druggists' Circular*.

